# Effect of an exosuit on daily life gait performance in individuals with incomplete spinal cord injury: a randomized controlled trial

**DOI:** 10.1186/s12984-026-01941-8

**Published:** 2026-03-13

**Authors:** L. Visch, B. E. Groen, A. C. H. Geurts, I. J. W. van Nes, N. L. W. Keijsers

**Affiliations:** 1 Department of Research, Sint Maartenskliniek, Nijmegen, The Netherlands; 2 Department of Sensorimotor Neuroscience, Donders Institute for Brain, Cognition and Behaviour, Radboud University, Nijmegen, The Netherlands; 3 Department of Rehabilitation, Donders Institute for Brain, Cognition and Behaviour, Radboud University Medical Center, Nijmegen, The Netherlands; 4 Department of Rehabilitation, Sint Maartenskliniek, Nijmegen, The Netherlands

**Keywords:** Incomplete spinal cord injury, Daily life gait performance, Exosuit, Assistive device

## Abstract

**Introduction:**

Individuals with incomplete spinal cord injury (iSCI) often have impaired gait capacity, limiting their gait performance at home and in the community. Lightweight lower extremity exosuits, such as the Myosuit (MyoSwiss AG, Zurich, Switzerland), may offer functional support to improve daily life gait performance. The aim of this randomized controlled trial was to investigate the effect of the Myosuit on daily life gait performance and its usability in individuals with iSCI.

**Methods:**

Thirty-four individuals with chronic iSCI were randomized (1:1) to either the intervention or control group. The intervention group received a Myosuit program consisting of five gait training sessions, followed by a 6-week home period in which the Myosuit was used as an assistive device. The control group received a conventional program consisting of four gait training sessions, followed by a 6-week home period without the device. Walking time per day was assessed, using an activity monitor, at baseline and during week one, three, and six of the home period. In addition, usability was assessed using the Dutch version of the System Usability Scale (D-SUS) and the Dutch version of the Quebec User Evaluation of Satisfaction with assistive Technology (D-QUEST) questionnaire after the home period.

**Results:**

No significant group difference in walking time per day during the home period was observed (estimate=9 min, robust standard error=8, p=0.23). The average D-SUS score was 56 ± 21 and the D-QUEST score was 3.4 ± 0.5.

**Conclusion:**

Myosuit use at home and in the community did not lead to improved daily life gait performance in individuals with iSCI. Usability was marginal, suggesting that further improvements of exosuit design and functionality are necessary to enhance its effectiveness and user acceptance in real-world settings.

*Trial registration* Clinicaltrials.gov NCT05605912. Registered on October 19, 2022.

**Supplementary Information:**

The online version contains supplementary material available at 10.1186/s12984-026-01941-8.

## Introduction

In individuals with an incomplete spinal cord injury (iSCI), the damage is partial preserving some motor, sensory, and autonomic functions below the level of injury [1]. Nevertheless, leg muscle weakness, sensory loss, and/or coordination impairments may significantly reduce gait capacity (i.e. what individuals are able to do in a standardized environment) [2–4]. This loss of gait capacity often results in reduced daily life gait performance (i.e. what individuals habitually do in daily life) [3–5]. Reduced daily life gait performance negatively influences personal independence and social interaction [6, 7]. It also increases the risk of secondary health complications including pain, bowel and bladder dysfunction, and pressure sores [8, 9]. Although passive assistive devices, such as crutches and walkers may provide support, individuals with iSCI often remain less ambulant [2].

Exoskeletons have undergone extensive development and hold promise as active assistive devices to improve gait capacity, thereby potentially enhancing daily life gait performance. The first generation were rigid exoskeletons primarily developed for individuals with no or limited gait capacity due to e.g. complete spinal cord injury. Rigid exoskeletons are able to completely take over control of the lower limbs. However, their heavy structure and limited walking speed make them less suitable for individuals with residual gait capacity [10]. More recently, soft exoskeletons (i.e. exosuits) have emerged as a next generation solution. These lightweight devices, composed of soft textiles with a minimum of rigid structures [11], are only suitable for individuals with residual gait capacity, such as individuals with iSCI, as they require substantial active muscle contribution from the users. Exosuits provide additional forces around the lower extremity joints to compensate for leg muscle weakness while following the user’s movements. Currently, rigid exoskeletons and exosuits are mainly used for gait training in clinical settings and are barely used at home [12], despite the fact that they hold potential for enhancing gait performance in home and community settings in individuals with iSCI.

A few studies evaluated the use of exosuits in home and community settings in individuals with neurological disorders [13–16]. Two studies investigated gait training with exosuits outside clinical settings in individuals with Parkinson’s disease and post stroke [13, 15]. When used for ambulation at home, exosuit (Myosuit) usability was rated as sufficient by individuals with diverse neurological disorders [16]. To date, only one study has evaluated the effect of home-based exosuit (Keeogo) use on gait performance in individuals with multiple sclerosis, yielding no significant difference in step count compared to a control group after two weeks [14]. The absence of an effect may be explained by limited familiarization as participants received only one session prior to home use and because the home period was relatively short. In addition, the findings of this study may not be generalized to other exosuits and patient populations. Therefore, studies are required to evaluate the effect of exosuits on gait performance in home and community settings in individuals with iSCI, incorporating adequate familiarization sessions and an extended home-use period. Based on previous literature, it can be expected that an exosuit (Myosuit) used as an assistive device improves gait capacity [17, 18]. As a consequence individuals may engage in more daily walking activities, thereby enhancing daily life gait performance (i.e., daily walking time). This is especially plausible because individuals with iSCI typically walk less due to their limited gait capacity. Furthermore, sufficient usability seems essential to enable meaningful improvements in daily life gait performance.

To this end, we performed a randomized controlled trial (RCT), in which participants assigned to the intervention group completed five exosuit training sessions in a specialized clinic, followed by a six-week home period of exosuit use. The control group received similar training sessions without the exosuit, followed by a six-week home period. The primary aim was to investigate the effect of an exosuit (Myosuit) used as an assistive device on daily life gait performance in individuals with iSCI. Because the exosuit is expected to improve assisted gait capacity [17, 18], we hypothesized that its use would increase daily walking time. In addition, we evaluated the usability of the exosuit in home and community settings. Since increased daily walking time may affect unassisted gait capacity and quality of life, we also evaluated the effect of a six-week home period of exosuit use on unassisted gait capacity as well as its short term impact on direct costs and quality of life.

## Methods

### Study design

A two-armed, open-label RCT was conducted at the Sint Maartenskliniek, Nijmegen, The Netherlands. A patient representative was actively involved in the study’s design phase. The study was approved by the internal review board of the Sint Maartenskliniek and the regional medical ethics committee Oost-Nederland (2022–13719, NL80641.091.22). The trial was registered in Clinicaltrials.gov (NCT05605912) and the study protocol was pre-published [19]. The study was conducted in accordance with the Declaration of Helsinki (64th WMA General Assembly, Fortaleza, Brazil, October 2024) and the Medical Research Involving Human Subjects Act. Reporting was according to the consolidated standards of reporting trials guidelines [20].

### Participants

Participants were recruited by rehabilitation physicians at the outpatient clinic of the Sint Maartenskliniek. In addition, recruitment occurred via the Dutch patient organization, where patients could apply for the study themselves. Inclusion criteria were: individuals with a spinal cord injury grade C or D according to the American Spinal Injury Association (ASIA) impairment scale [21], time since injury > 6 months, age 18 years and older, sufficient hand function to don and doff the exosuit (or access to a caregiver who could assist with donning and doffing at home), reduced gait capacity due to reduced knee and/or hip strength (Medical Research Council (MRC) scale < 5) [22], ability to rise from a chair without deviating more than 45° to either side, ability to walk independently for 10 m with or without passive assistive devices, and a personal aim to improve walking distance, speed, or gait capacity. Participants were excluded if they: had a concurrent (neurological) condition affecting motor performance, had wounds that could be worsened by wearing the exosuit, had a body height < 150 cm or > 195 cm or body weight < 45 kg or > 110 kg, were pregnant, had a flexion contracture at the knee or hip joint > 10°, or had varus or valgus deformity at the knee > 10°. Participants provided written informed consent before the start of the study.

### Exosuit

**Fig. 1:**
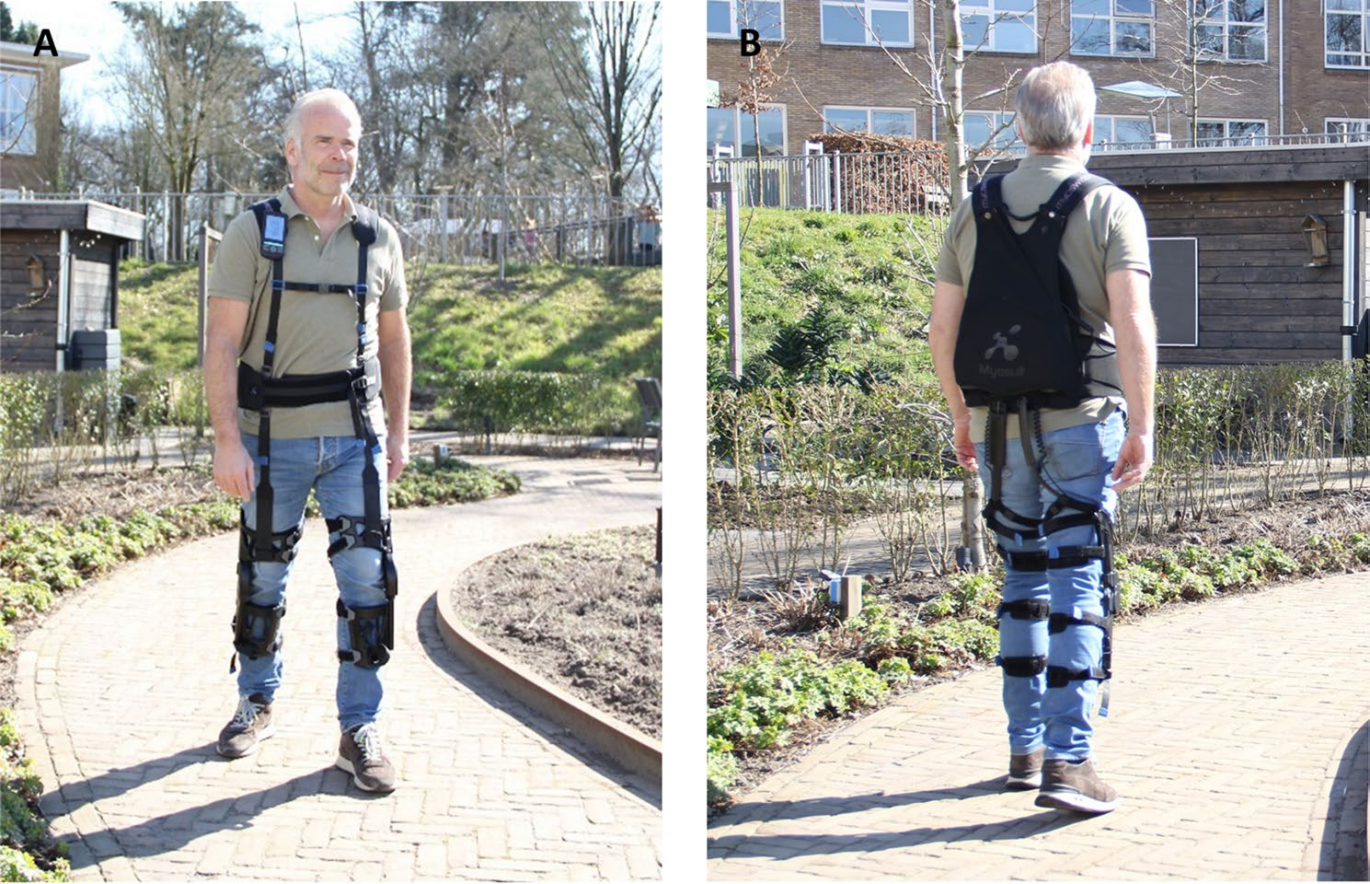
Myosuit. Front view (**A**). Back view (**B**)

The exosuit used in this study was the Myosuit (MyoSwiss AG, Zurich, Switzerland; weight 5.5 kg; see Fig. 1) [23]. It provides active hip and knee extension support during standing and walking by Bowden cables running behind the legs, behind the hip and in front of the knee joints. In the standing mode, the cables are constantly tensioned. During walking, the cables are tensioned during the stance phase of the gait cycle. Assistance levels range from 0 (minimal support) to 5 (maximum support) and can be adjusted per leg. Users can switch between modes using a remote control attached to the shoulder strap. In addition, a polymer spring in front of the legs provides passive hip flexion support during the swing phase. An optional passive foot lifter can be attached to the shoe(s) to support foot clearance.

### Procedures

**Fig. 2:**
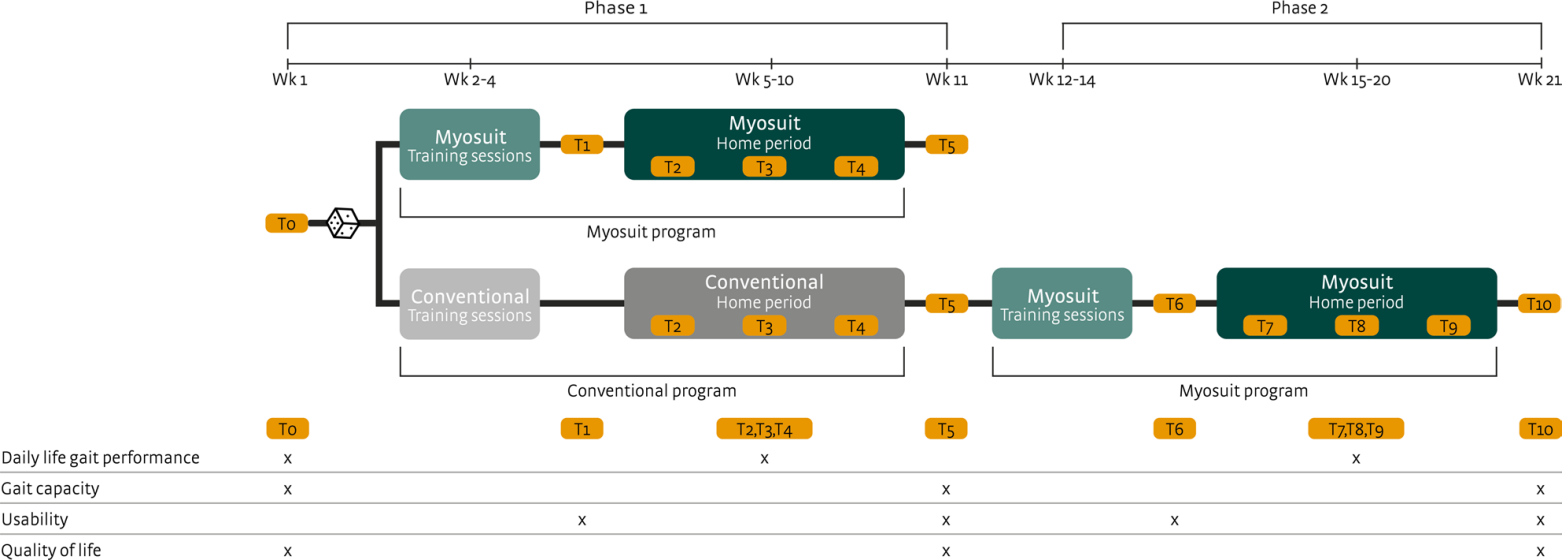
Schematic overview of the study design

A schematic overview of the study is shown in Fig. 2. Participants were randomized (using CastorEDC (www.castoredc.com)) in a 1:1 ratio to the intervention group or to the control group using block randomization with variable block sizes of 2 and 4. In addition, stratification was applied based on the preferred walking speed at baseline (< 0.6 m/s versus ≥ 0.6 m/s). The first study phase involved a RCT in which the intervention group received the Myosuit program and the control group received the conventional program. The Myosuit program consisted of a 3-week training phase followed by a 6-week home period of Myosuit use. The conventional program consisted of a similar training phase without the Myosuit, followed by a 6-week home period. In the second study phase, after completion of the conventional program, participants in the control group were subsequently enrolled in the same Myosuit program as the intervention group, thereby ensuring that all participants had access to the Myosuit. During the entire study period, all participants were allowed to continue with usual care (e.g. physiotherapy).

Blinding of the participants and treating physical therapists was not feasible due to the nature of the intervention. The primary researcher (LV) was responsible for the randomization procedure and for all assessments. Therefore, outcome assessments could not be blinded either. At baseline (T0), the recorded participant characteristics included sex, age, time since injury, height, weight, injury level according to the ASIA impairment scale, level of spinal cord injury, leg muscle strength (MRC scale), leg muscle tone (Ashworth scale), somatosensation of the lower limbs (ASIA impairment scale), WISCI II (Walking Index for Spinal Cord Injury) score, and SCIM III (Spinal Cord Independence Measure) mobility score. During the first study phase, assessments were performed at baseline (T0), after the training sessions (T1), during the first, third, and sixth week of the home period (T2, T3, T4), and post-intervention (T5). During the second study phase, additional assessments were conducted for the control group at similar time intervals (T6, T7, T8, T9, T10).

### Interventions

#### Myosuit program

The Myosuit program consisted of five individual training sessions led by a physical therapist, scheduled across a 3-week training period, each lasting 60 to 90 min, followed by a 6-week home period during which participants could use the Myosuit themselves. The training sessions consisted of two parts: learning independent Myosuit operation, including donning, doffing, and using the remote control (2.5 h total; 5 sessions of 30 min each), and performing standing and walking exercises with the Myosuit (4 h total; 3 sessions of 1 h and 2 sessions of 30 min). These exercises, detailed in a previously published protocol paper [19], were tailored to the daily activities that participants would like to perform with the Myosuit at home and in the community. After the training sessions, participants completed a test to obtain a Myosuit certificate for independent home use. Thereafter, participants received the Myosuit to independently use it at home. In addition, they received an information sheet with general exercise recommendations for individuals with spinal cord injury.

#### Conventional program

The conventional program consisted of individual training sessions led by a physical therapist, followed by a 6-week home period. To ensure that the conventional program received a similar amount of exercise time (4 h total) and to reduce the burden of clinic visits, the sessions were scheduled as four sessions of 60 min over a 2-week period. Exercises focused on standing and walking were similar to the Myosuit program, and adjusted to the participants’ daily activities at home and in the community. After the training sessions, participants received an information sheet with general exercise recommendations for the 6-week home period, tailored to individuals with spinal cord injury.

### Outcome measures

#### Primary outcome measure

##### Daily life gait performance

The primary outcome measure for daily life gait performance was walking time per day (min), assessed with an activity monitor (Activ8 Basic Activity Tracker, Activ8, Valkenswaard, The Netherlands) [24]. The intervention group wore the Acitv8 sensor for seven consecutive days (24 h a day) on their right upper leg at T0, T2, T3, and T4. The control group also wore the Activ8 sensor for seven consecutive days (24 h a day) on their right upper leg at T0, T2, T3, and T4 as well as at T7, T8, and T9. Baseline data were calculated as the mean across all available complete days (T0). For the 6-week home period, data from all measurements (T2-T4 or T7-T9) were averaged across all available complete days.

#### Secondary outcome measures

##### Usability

During the home period, participants completed a daily logbook documenting Myosuit usage and activity type. The outcome measures were frequency and purpose of use. Usability and satisfaction were assessed with the Dutch version of the System Usability Scale (D-SUS) [25] and the Dutch version of the Quebec User Evaluation of Satisfaction with assistive Technology (D-QUEST) [26]. Participants in the intervention group completed these questionnaires at T1 and T5, while participants in the control group completed these questionnaires at T6 and T10. The D-SUS assesses usability with scores ranging from 0 to 100: 0–50 indicating “not acceptable”, 51–67 “marginal usability”, and 68–100 “acceptable usability”. The D-QUEST assesses satisfaction with 12 items ranging from 1 (“totally dissatisfied”) to 5 (“very satisfied”). It provides an overall satisfaction score (sum of 12 items), and two subscale scores: assistive device (8 items) and service provided (4 items). For these three scores, the averages were calculated. In addition, participants selected the three most important items, for which an item score of 4–5 was considered as satisfied, and an item score of 1–3 was considered as not satisfied.

##### Gait capacity

Gait capacity was evaluated with the 10-Meter Walk Test at preferred and maximum walking speed (m/s) [27], the 6-min Walk Test as a measure of walking distance (m) [28], and the Spinal Cord Injury Functional Ambulation Profile (SCI-FAP) (lower scores reflecting better gait capacity) [29]. The intervention group completed these assessments at T0 and T5, while the control group completed them at T0, T5, and T10. Passive assistive devices were kept constant across all time points.

##### Costs and quality of life

The actual costs related to the Myosuit intervention (purchase costs, maintenance costs, and costs related to the training sessions provided by a physical therapist [30]) and to the conventional program (costs related to training sessions provided by a physical therapist [30]) were calculated. Quality of life was assessed at T0, T5, and T10 using the EuroQol 5-Dimension 5-Level (EQ-5D-5 L) questionnaire [31]. Based on the EQ-5D-5 L, the utility index and quality adjusted life years (QALYs) gained were calculated [32]. The utility index ranges from 1 (representing full health) to 0 (representing death). QALYs gained were calculated by multiplying the utility index by years lived in that state [31], which was assumed to be one year in our study. For each group, differences in QALYs gained between T0 and T5 were calculated. For the control group, also differences in QALYs gained between T5 and T10 were calculated.

##### Spatio-temporal gait characteristics and the general and disease-specific self‑efficacy scale (GDSSeS)

Results for spatiotemporal gait parameters as originally planned (see protocol paper [19]) were not reported, because a self-developed algorithm, although validated in patients with neurological disorders [33], appeared unreliable for detecting gait events in our study participants, likely due to their slow walking speed. Similarly, the originally planned GDSSeS was not used in the current study because it had not yet been validated.

### Sample size

An improvement of 500 steps per day was considered clinically relevant. This corresponded to a 33% increment of a baseline with 1500 steps per day [34]. 500 steps per day corresponds to an increase of 4.5 min walking time per day, based on a stride time of 1.07 s [35]. A sample size of *n* = 14 was needed for each group to show a group difference of 500 steps per day (standard deviation = 400, *α* = 0.05, *β* = 0.10) [34]. To allow an 18% attrition rate, three patients were added to each group (*n* = 17). Therefore, we included 34 participants [19].

### Statistical analysis

An intention-to-treat analysis was performed for all RCT outcomes, with missing data handled using the last observation carried forward method. This method implies that missing values were replaced with the last available measurement. All analyses were performed in R (version 2022.12.0) with a significance level of 0.05. For secondary analyses, data from the first study phase of the intervention group and of the second study phase of the control group were used. Mean differences between time points were calculated for each group, and the responses of both groups to the Myosuit intervention were compared descriptively.

#### Primary outcome measure

Group differences in walking time per day during the home period (T2-T4) were analyzed by Analysis of Covariance (ANCOVA), using baseline values (T0) as covariates. Although residuals were not normally distributed, the proportion of standardized residuals above 2 or 3 did not exceed 10% or 1% of the data, respectively. Therefore, an ANCOVA remained valid [36]. However, due to violated homoscedasticity, the Wald test using heteroscedasticity-robust standard errors was performed [37].

In the secondary analyses, the aim was to examine whether specific characteristics (age, lesion level, ASIA impairment scale, time since injury, WISCI II score, somatosensation, and exosuit home use) predicted changes in walking time while using the Myosuit at home, using a regression analysis. To justify pooling the data from the Myosuit programs of both groups, we first calculated the mean differences and 95% confidence intervals (CIs) in walking time per day between baseline and home periods (intervention group: T0 vs. T2-T4; control group: T4 vs. T7-T9) for each group. After this step, the regression analysis was performed.

#### Secondary outcome measures

For usability, frequency and purpose of use, D-SUS scores, and D-QUEST scores for the intervention group (T1 and T5) and the control group (T6 and T10) were reported descriptively.

For the effect of the intervention on gait capacity (preferred and maximum walking speed and walking distance) an ANCOVA was performed to test group differences post-intervention (T5) with baseline values (T0) as covariates. Group differences in SCI-FAP score post-intervention (T5) were analyzed using the Wald test using heteroscedasticity-robust standard errors, due to violated homoscedasticity, with baseline values (T0) as covariates. As a secondary analysis, mean differences and 95% CIs in capacity measures from baseline to post-intervention (intervention group: T0 vs. T5; control group: T5 vs. T10) were calculated.

Costs and QALYs gained (T0 vs. T5) were analyzed descriptively. As a secondary analysis, QALYs gained for the control group (T5 vs. T10) were reported as well.

## Results

**Fig. 3:**
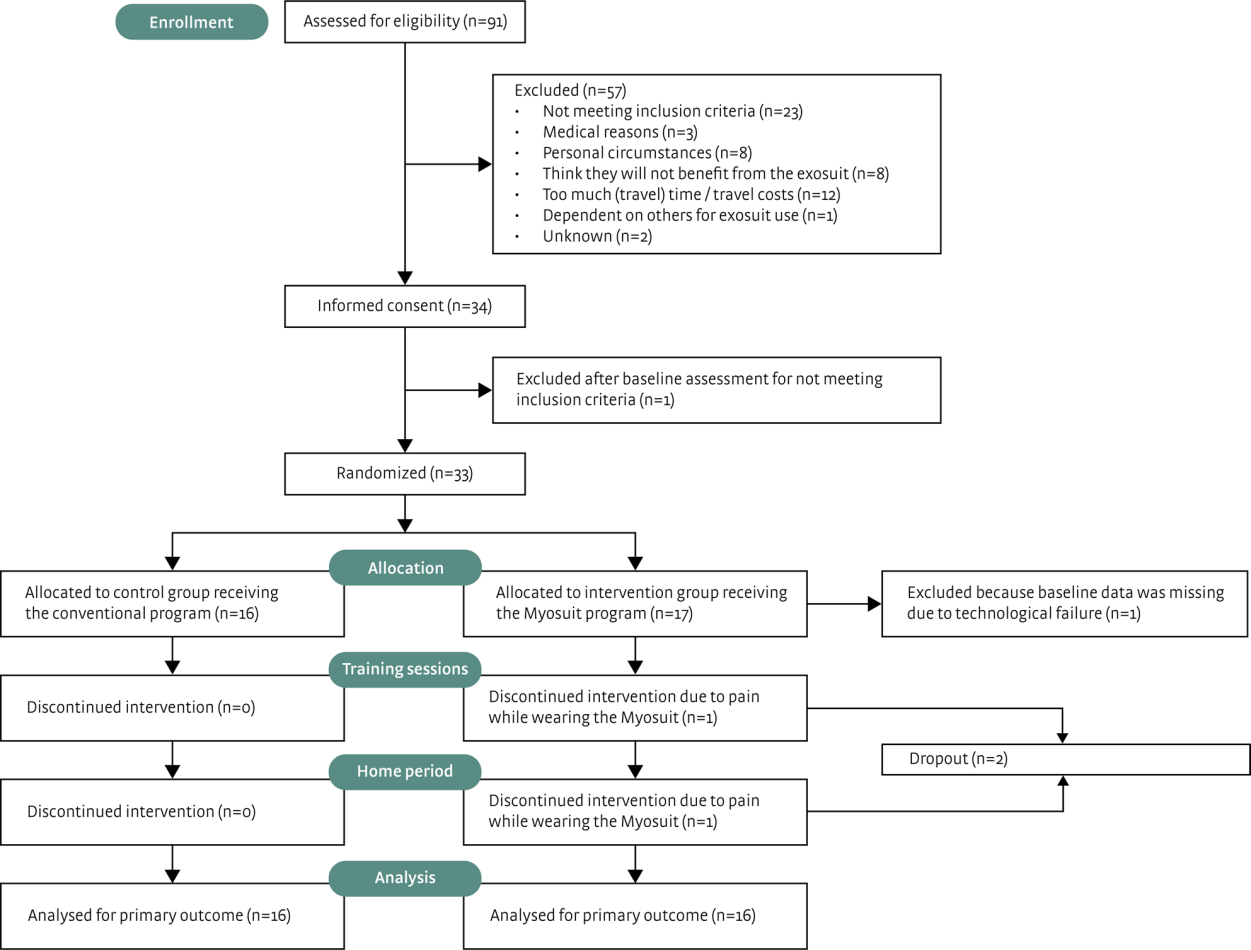
Flow diagram of participants

Participants were enrolled between October 2022 and August 2025. Of the 34 participants who provided written informed consent, one was excluded during baseline assessment for not meeting the inclusion criterium “ability to walk independently for 10 m”. From the remaining 33 participants, 16 were allocated to the control group receiving the conventional program and 17 to the intervention group receiving the Myosuit program (see Fig. 3). In the intervention group, one participant was excluded because of missing baseline data of the primary outcome measure due to a technological failure. Two participants dropped out during the study due to pain while using the Myosuit (one during the training sessions and one in the first week of the home period). Following the intention-to-treat approach these participants were included in the primary analysis (first study phase) for the primary outcome. Consequently, 16 participants in the intervention group and 16 participants in the control group were included. Baseline participant characteristics are shown in Table 1, demonstrating that no meaningful group differences were present. One participant in the intervention group did not meet a specific inclusion criterion (grade C or D according to the ASIA impairment scale), but was nevertheless included based on sufficient gait capacity.

**Table 1:** Participant characteristics^a^

Demographic	Intervention group (Myosuit program) *n* = 16	Control group (conventional program) *n* = 16
Age (y)^b^	57 ± 14	61 ± 16
Weight (kg)^b^	79 ± 15	80 ± 13
Height (cm)^b^	179 ± 9	177 ± 8
Sex		
Male	9	10
Female	7	6
Injury-related		
ASIA impairment scale		
Grade B	1	0
Grade C	5	5
Grade D	10	11
Injury level		
Cervical	5	8
Thoracic	5	7
Lumbar	6	1
Time since injury (y)^b^	11 ± 10	10 ± 9
Cause		
Traumatic	9	7
Non-traumatic	7	9
Muscle strength^b^	33 ± 11	38 ± 9
Muscle tone^b^	3 ± 5	7 ± 6
Somatosensation^b^	55 ± 21	49 ± 18
Mobility		
WISCI II^c^	13 (9–20)	14 (4–20)
SCIM III mobility^c^	16 (8–29)	17 (6–29)

*ASIA* American Spinal Injury Association, *WISCI II* Walking Index for Spinal Cord Injury, *SCIM III* Spinal Cord Independence Measure

^a^ Presented as number of participants unless indicated otherwise

^b^ Presented as mean ± SD

^c^ Presented as median (range)

Muscle strength is the total motor score for the five key lower extremity muscles of both legs according to the ASIA Impairment Scale, based on the Medical Research Council scale (range 0 to 50)

Muscle tone is to total score for knee flexors and extensors, and ankle plantar flexors and dorsiflexors of both legs, based on the Modified Ashworth Scale (range 0 to 32)

Somatosensation is the total score for the dermatomes from T10 to the most caudal level for pin prick and light touch sensation of both sides (range 0 to 96)

WISCI II indicates the ability to walk and is the preferred WISCI II level (range 0, indicating most severe level of disability, to 20, indicating minimal impairment)

SCIM III mobility is the sum of the five indoor and outdoor mobility questions: indoors, moderate distance, outdoors, stair management, transfer wheelchair-car, transfer ground-wheelchair (range 0–30)

### Primary outcome measure

#### Daily life gait performance

**Fig. 4:**
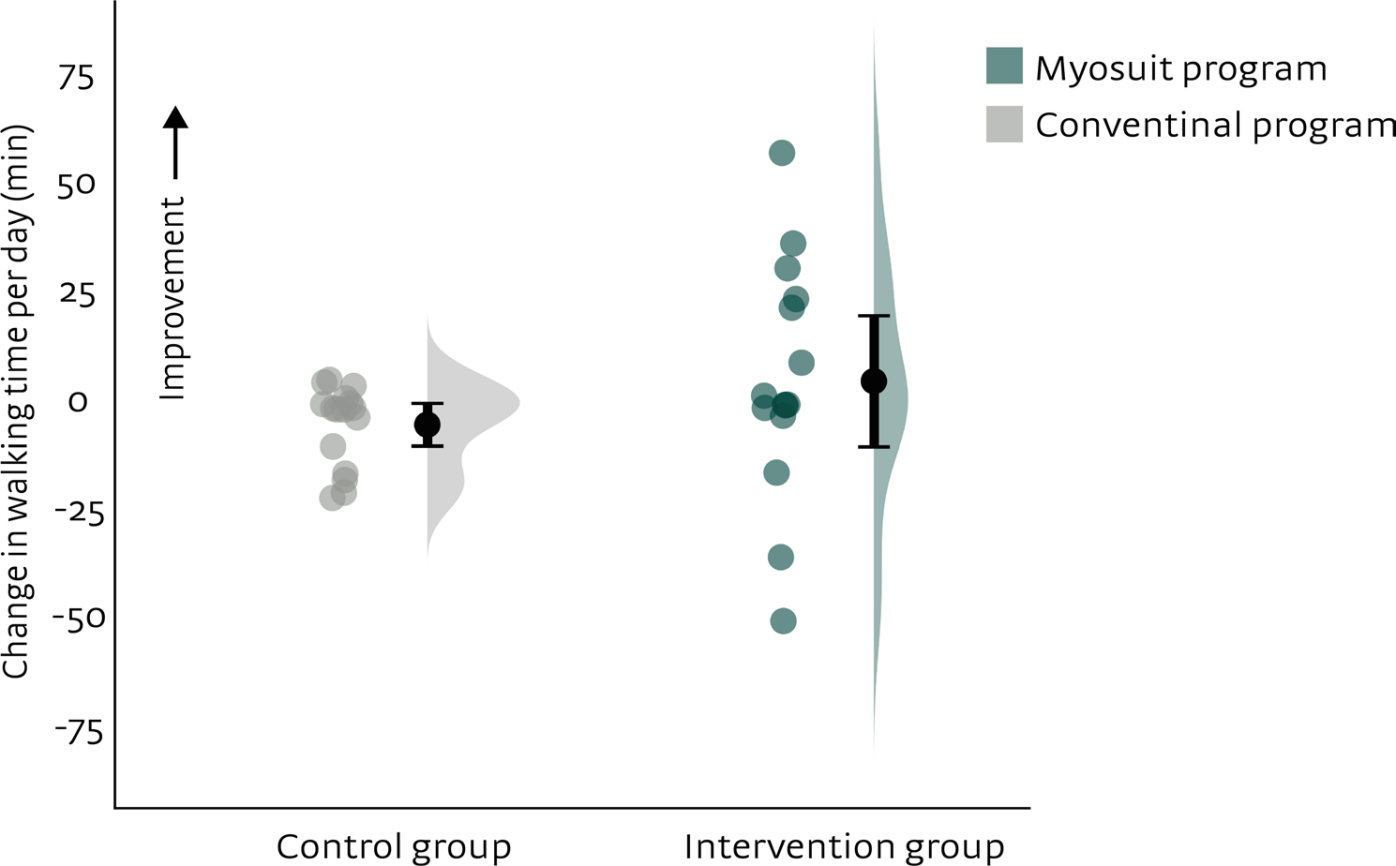
Individual differences in daily life gait performance during the home period (average T2-T4) with respect to baseline (T0). Dots represent individual data points and bars the means and 95% confidence intervals

Results for walking time per day are shown in Fig. 4. No significant group differences in walking time per day during the home period (T2-T4), corrected for baseline values (T0), were found (robust standard error = 8, *p* = 0.23) (see Table 2). The mean group difference was 9 min (Wald confidence interval=−6, 25).

**Table 2:** Daily life gait performance^a^

	Intervention group (Myosuit program) *n*=16		Control group (conventional program) *n*=16		Mean group difference^b^	Wald test
	T0	Average T2-T4	T0	Average T2-T4	Average T2-T4	
Walking time per day (min)	59 ± 59	63 ± 58	64 ± 51	58 ± 46	9 (−6, 25)	Robust SE=8, *p*=0.23

*SE* standard error

^a^ Presented as mean ± SD unless indicated otherwise

^b^ Presented as mean (Wald confidence interval): mean group difference during the home period (average T2-T4) (intervention group – control group) adjusted for group differences at baseline (T0)

### Secondary analyses

The 95% CIs of the mean changes in walking time per day from baseline to home period for the intervention and the control group are shown in Supplementary material 1, indicating a clear overlap between the responses of both groups. In addition, participants showed no significant associations of personal characteristics with changes in walking time per day (see Supplementary material 2).

### Secondary outcome measures

#### Usability

**Fig. 5:**
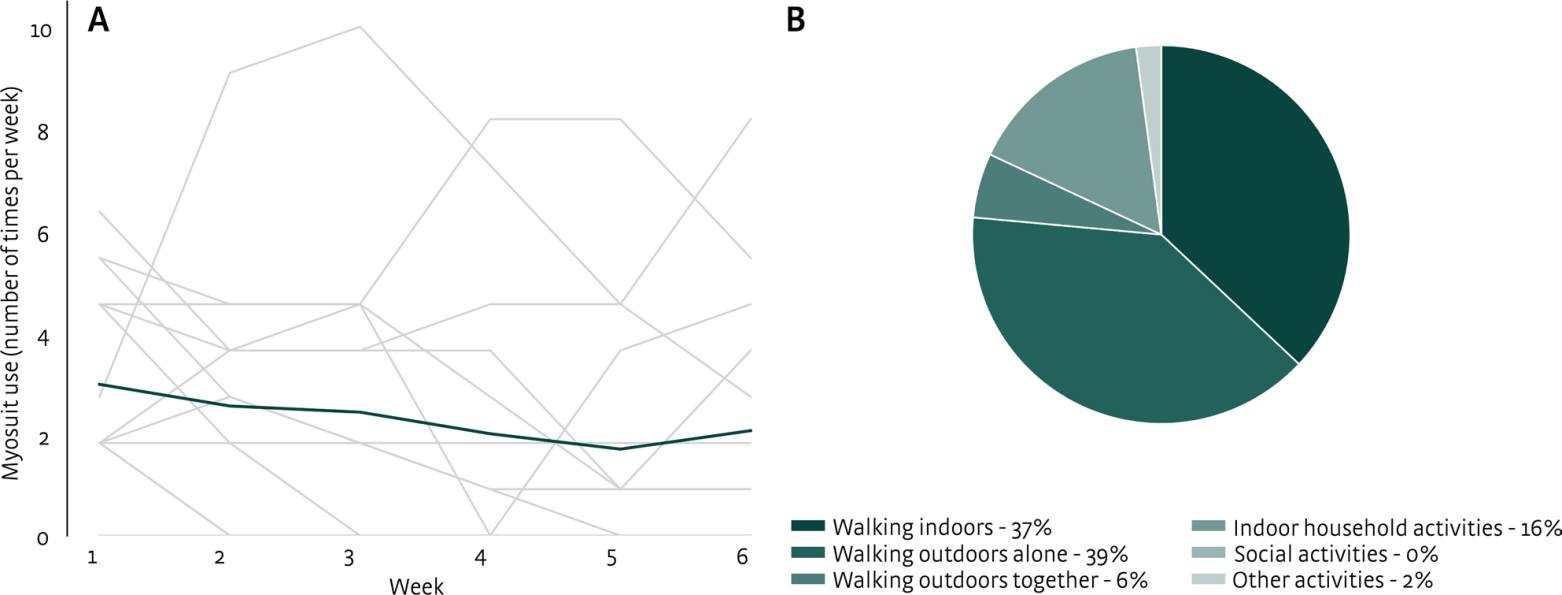
Myosuit use of the intervention group following the Myosuit program (first study phase). Frequency of use per week (**A**). Grey lines represent individual use, and the dark green line indicate the mean use per week. Activities during which the Myosuit was used (**B**)

Participants in the intervention group used the Myosuit on average 2.7 ± 2.4 times per week during the 6-week home period (see Fig. 5A). They mainly used the Myosuit for walking indoors and walking outdoors on their own (see Fig. 5B). The average score on the D-SUS was 64 ± 12 at T1 and 56 ± 21 at T5, indicating marginal usability at both time points. At T1, the average satisfaction was rated as 3.5 ± 0.4 (total D-QUEST score), with subscale scores assistive device 3.4 ± 0.4 and service 3.7 ± 0.5. The most frequently selected important items were: effectiveness (*N* = 6 dissatisfied, *N* = 9 satisfied), ease of use (*N* = 11 dissatisfied, *N* = 4 satisfied), and weight (*N* = 10 dissatisfied, *N* = 5 satisfied). At T5, the average satisfaction with the Myosuit was rated as 3.4 ± 0.5 (total D-QUEST score), with subscale scores assistive device 3.1 ± 0.7 and service 3.9 ± 0.5. The most important items were: effectiveness (*N* = 8 dissatisfied, *N* = 7 satisfied), ease of use (*N* = 12 dissatisfied, *N* = 3 satisfied), and comfort (*N* = 13 dissatisfied, *N* = 2 satisfied). Myosuit use, D-SUS scores, and D-QUEST scores of the control group are reported in Supplementary material 3.

#### Gait capacity

No significant group differences in any gait capacity measure post-intervention (T5), corrected for baseline values (T0), were found (see Table 3). Supplementary material 4 presents individual differences (T0 vs. T5) in each outcome measure. Secondary, the 95% CIs of the mean changes in gait capacity measures from baseline to post-intervention for the intervention and the control groups are shown in Supplementary material 5. These secondary analyses showed substantially overlapping responses of both groups.

**Table 3:** Gait capacity measures^a^

	Intervention group (Myosuit program) *n*=15		Control group (conventional program) *n*=16		mean group difference^b^	ANCOVA
	T0	T5	T0	T5	T5	
Preferred walking speed	0.57 ± 0.27	0.66 ± 0.33	0.55 ± 0.25	0.61 ± 0.28	0.02 (−0.03, 0.06)	*F*(1, 30)=0.54, *p*=0.57
Maximum walking speed	0.73 ± 0.36	0.80 ± 0.40	0.69 ± 0.30	0.74 ± 0.34	0.02 (−0.03, 0.06)	*F*(1, 30)=0.47, *p*=0.51
Walking distance	211 ± 132	240 ± 134	198 ± 99	204 ± 108	12 (−2, 25)	*F*(1, 30)=2.92, *p*=0.10
					mean group difference^c^	Wald test
SCI-FAP	287 ± 347	286 ± 349	299 ± 510	286 ± 503	−6 (−13, 2)	Robust SE=4, *p*=0.15

*ANCOVA* Analysis of Covariance, *SCI-FAP* Spinal Cord Injury Functional Ambulation Profile, *SE* standard error

^a^ Presented as mean ± SD unless indicated otherwise

^b^ Presented as mean (95% confidence interval): mean group difference post-intervention (T5) (intervention group – control group) adjusted for group differences at baseline (T0)

^c^ Presented as mean (Wald confidence interval): mean group difference post-intervention (T5) (intervention group – control group) adjusted for group differences at baseline (T0)

#### Costs and QoL

Costs per patient for the Myosuit program were €505.57 for the training sessions, €6,810 for the purchase of the Myosuit, and €5,600 for five-year maintenance of the Myosuit. Cost per patient for the conventional program were €311.12 for the training sessions. For quality of life, the mean differences in QALYs gained were 0.06 ± 0.10 for the intervention group and 0.03 ± 0.15 for the control group (T0 vs. T5). The cost per QALY for the Myosuit program was €203,075 and for the conventional program was €9,457. QALYs gained for the control group during the second study phase are reported in Supplementary material 6.

### Adverse events and device

One serious adverse event (SAE) and eight adverse events (AEs) occurred during the study. The SAE occurred during the conventional program, was unrelated to the intervention, and did not lead to discontinuation. All AEs occurred during the Myosuit program: six in the intervention group (first study phase) and two in the control group (second study phase). In the intervention group, three AEs were related to the Myosuit (one fall without severe consequences and two cases of pain leading to discontinuation of the program: one after the first training session and one during the first home week). The other three were unrelated to the Myosuit (one case of pressure ulcer on the buttock caused by a warm jug and two skin irritations from the activity monitor). In the control group, one AE was related to the Myosuit (a fall without severe consequences) and one was unrelated to the Myosuit (concerns about pacemaker), with the latter leading to discontinuation during the first week of the home period.

Of the 33 participants who used the Myosuit, 14 used at least one foot lifter. During the Myosuit program, a total of thirteen device errors occurred: four in the intervention group (first study phase), six in the control group (second study phase), and three unrelated to specific participants. In the intervention group, two occurred during the training sessions and two during the home period: one on the first day, which delayed measurements by one week, and one near the end, which prevented use during the final four days. In the control group, two device errors occurred during the training sessions and four at home, causing interruptions of Myosuit use from a few days to up to four weeks.

## Discussion

This is the first study that investigated the effect of an exosuit on daily life gait performance in individuals with iSCI. While we hypothesized that exosuit-assisted walking would enhance daily walking time, no significant difference was found between the intervention and control group. Our findings align with a previous RCT in individuals with multiple sclerosis that reported no changes in daily step count while using an exosuit at home for two weeks [14]. Even though our study, in contrast to the former one, included multiple supervised familiarization sessions, implemented a home use period of six weeks, and used an exosuit supporting both hip and knee control (i.e. Myosuit) rather than only the knee (i.e. Keeogo), no improvements were observed in daily walking time [14]. Importantly, in our study the change in daily walking time during the home period compared to baseline showed greater variability in the intervention group than in the control group. Some participants improved whereas others declined in their performance, suggesting that exosuit use may offer benefits for a subset of individuals. Regrettably, secondary analyses did not reveal specific characteristics that could predict individual responses. As a result, no implications for future stratified interventions could be drawn, underscoring the need for further research to better understand the factors that influence responsiveness to exosuit interventions.

The observed absence of beneficial effects of exosuit use on daily life gait performance in individuals with iSCI can be interpreted within the framework of the Technology Acceptance Model [38]. Within this model, perceived ease of use and perceived usefulness are key determinants of behavioral intention and actual device use [38]. In our study, exosuit usage varied considerably, aligning with previous studies on both exosuits and rigid exoskeletons showing inconsistent adherence across users [16, 39, 40]. Perceived ease of use with the exosuit appeared low, as reflected by marginal usability and satisfaction scores. Compared to previous research investigating exosuit usability in individuals with neurological disorders, our participants reported lower scores for both usability and satisfaction (average SUS score of 56 versus 75, and an average QUEST assistive device score of 3.1 versus 4.1, respectively) [16]. This discrepancy may stem from differences in user expectations, as participants in the previous study were private purchasers [16], who may have held a more positive attitude towards the device. Furthermore, in our study several device errors occurred during training and home use, likely undermining participants’ trust in the exosuit and their perceived ease of use. The low perceived ease of use may have negatively affected the intention to use the exosuit, thereby limiting its actual use and impact on daily life gait performance.

In this study, perceived usefulness refers to the extent to which individuals with iSCI considered the exosuit beneficial for enhancing their daily life gait performance. This was captured by the D-QUEST item on effectiveness, which evaluates whether the device fulfils its intended purpose. Ratings demonstrated modest satisfaction with the exosuit’s impact on daily life gait performance. This result aligns with a previous study from our group [41], which showed that gait capacity while using an exosuit (i.e. Myosuit) did not exceed unassisted walking. This suggested that the anticipated improvement in gait capacity with an exosuit could not be confirmed, and consequently actual use and daily life gait performance may be limited.

A strength of this study is that participants followed multiple training sessions to familiarize themselves with the exosuit prior to the 6-week home use period. In addition, they were instructed to use the exosuit as an assistive device for personal daily activities, without a prescribed minimum duration or frequency. This approach aimed to capture the real-world impact of exosuit use rather than the effects of a structured activity regimen. A limitation that may have influenced the results was the heterogeneity of the study population, which may have contributed to variable responses to the Myosuit. Secondly, to identify whether specific characteristics predicted changes in daily walking time, a regression analysis was performed using pooled data from both groups, combining measurements taken at different time points, which may have introduced a temporal bias. Third, incomplete data for the primary outcome measure (daily walking time) may have affected our findings. Daily walking time could not always be calculated over seven full days for each assessment due to missing data. Instead, daily walking time was derived from the available days of each assessment period, which may have produced a less accurate estimate. Furthermore, daily life walking time does not entirely reflect daily life gait performance, as walking the same distance in less time also indicate functional improvement. It seems unlikely, however, that this consideration is relevant for interpreting the results of the present study, given a previous study from our group that found no effects of exosuit-assisted walking on gait capacity [41]. Other limitations were the reliance on self-reported home use via logbooks rather than device recorded data, potential selection bias towards participants with high interest in technology, and the absence of assessor blinding. Yet, the last two factors would be expected to influence the results in favor of the exosuit intervention, whereas no positive effect was observed.

## Conclusion

Our findings indicate that using an exosuit supporting both hip and knee extension in home and community settings does not improve daily life gait performance, assessed as walking time per day, in individuals with iSCI. The usability of the current exosuit (Myosuit) seems to be insufficient to improve daily life gait performance. Based on these results, routine prescription of an exosuit to enhance gait performance in home and community settings for individuals with iSCI is currently not supported.

## Supplementary Information

Supplementary Material 1

Supplementary Material 2

Supplementary Material 3

Supplementary Material 4

## Data Availability

The datasets used during the current study are available from the corresponding author on reasonable request.

## Abbreviations

AE: Adverse event
ANCOVA: Analysis of Covariance
ASIA: American Spinal Injury Association
CIs: Confidence intervals
D-SUS: Dutch version of the System Usability Scale
D-QUEST: Dutch version of the Quebec User Evaluation of Satisfaction with assistive Technology
EQ-5D-5L: EuroQol 5-Dimension 5-Level
GDSSeS: General and disease-specific self‑efficacy scale
iSCI: Incomplete spinal cord injury
MRC: Medical Research Council
QALYs: Quality adjusted life years
RCT: Randomized controlled trial
SAE: Serious adverse event
SCI-FAP: Spinal Cord Injury Functional Ambulation Profile
SCIM: Spinal Cord Independence Measure
WISCI: Walking Index for Spinal Cord Injury
