# Supplementary figures and images for: Effect of an exosuit on daily life gait performance in individuals with incomplete spinal cord injury: a randomized controlled trial
Source: J Neuroeng Rehabil. 2026 Mar 13;23:134. doi: 10.1186/s12984-026-01941-8 (PMC13101325; doi:10.1186/s12984-026-01941-8)

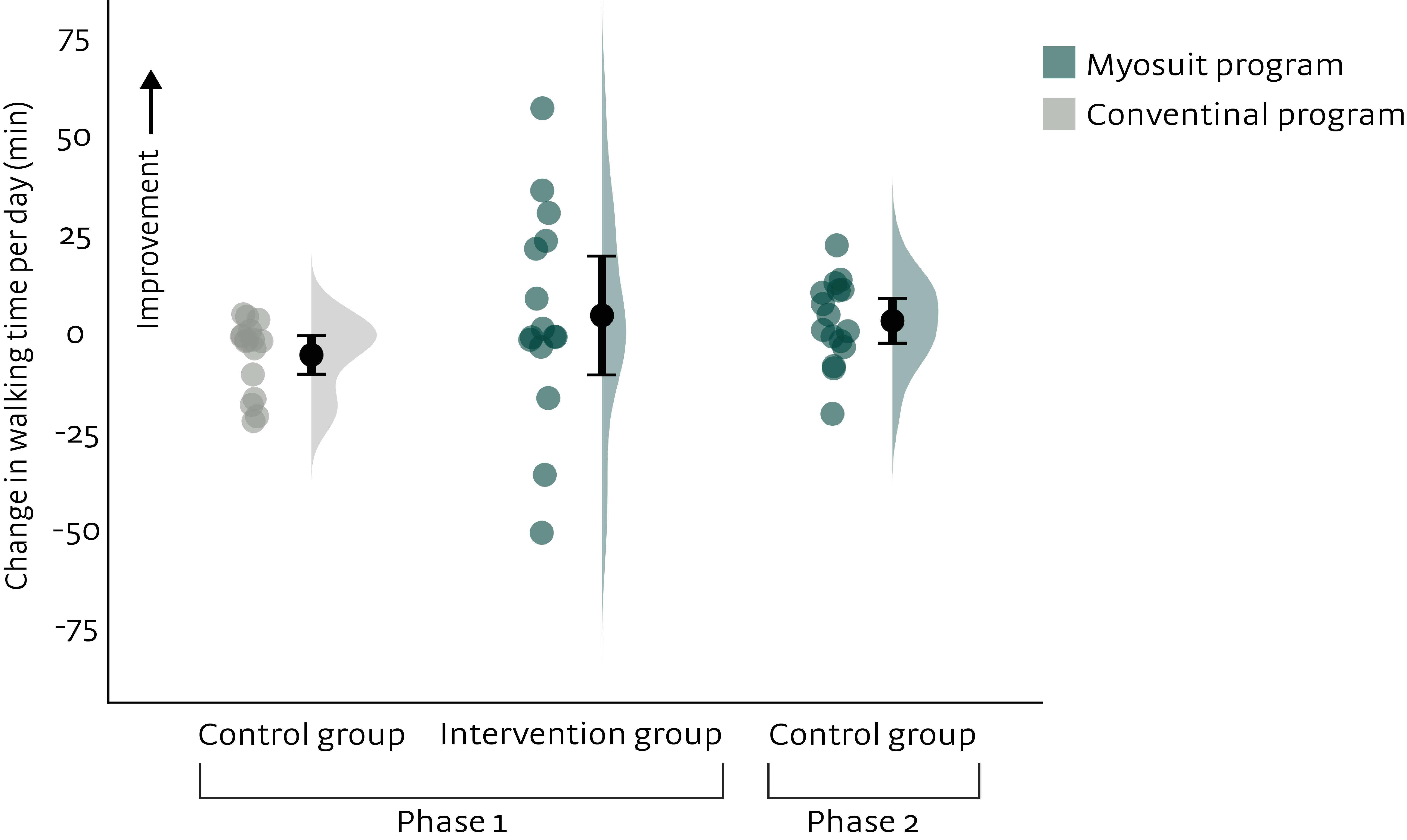

Supplement: Supplementary file 1 — Supplementary Material 1 [file 12984_2026_1941_MOESM1_ESM.png]

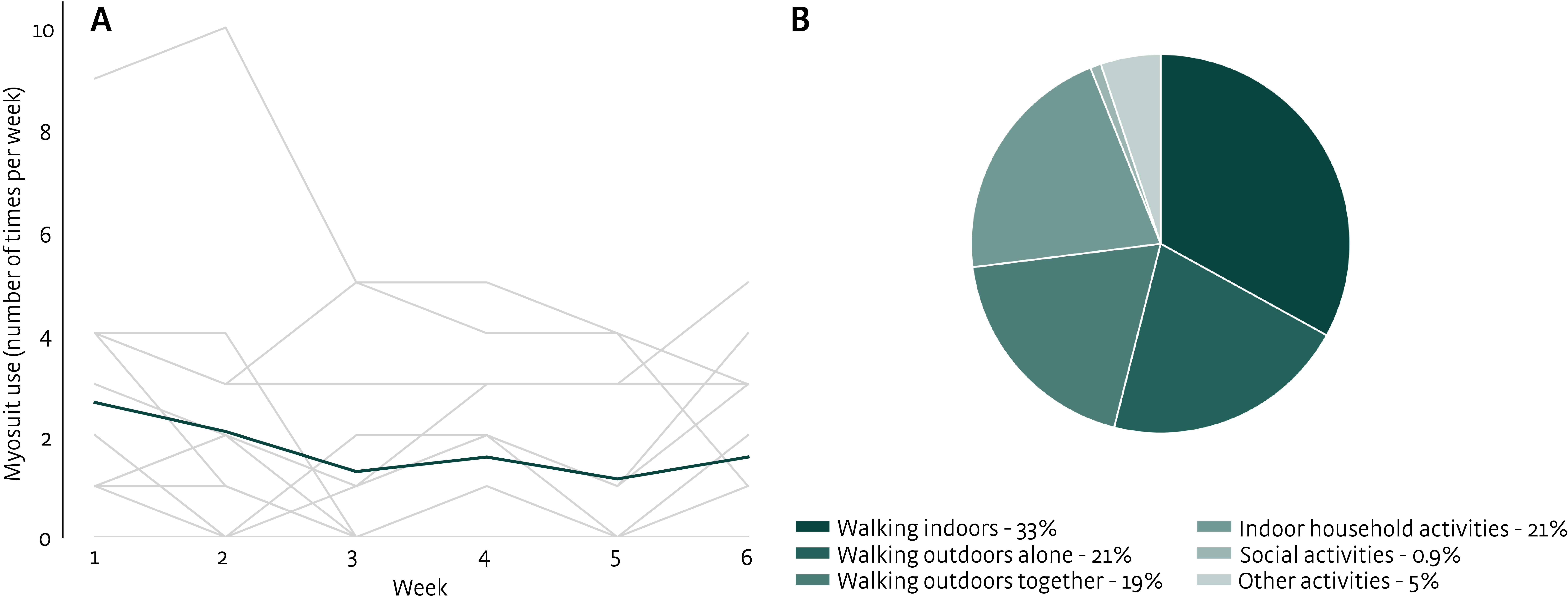

Supplement: Supplementary file 3 — Supplementary Material 3 [file 12984_2026_1941_MOESM3_ESM.png]
