# Supplementary material for: Effect of an exosuit on daily life gait performance in individuals with incomplete spinal cord injury: a randomized controlled trial
Source: J Neuroeng Rehabil. 2026 Mar 13;23:134. doi: 10.1186/s12984-026-01941-8 (PMC13101325; doi:10.1186/s12984-026-01941-8)
Supplement: Supplementary file 4 — Supplementary Material 4 [file 12984_2026_1941_MOESM4_ESM.docx]

**Supplementary material 1.** Secondary analyses primary outcome measure.

**Table SM1.** Daily life gait performance.^a^

|  | Intervention group (Myosuit program)  n = 16 | | | Control group (Myosuit program)  n = 16 | | |
| --- | --- | --- | --- | --- | --- | --- |
|  | T0 | Average T2-T4 | Difference^b^ | T4 | Average T7-T9 | Difference^b^ |
| Walking time per day (min) | 59 ± 59 | 63 ± 58 | 4 (-9, 17) | 57 ± 47 | 60 ± 45 | 3 (-2, 8) |

^a^ Presented as mean ± SD unless indicated otherwise

^b^ Presented as mean (95% confidence interval): difference between the home period (average T2-T4 for the intervention group and average T7-T9 for the control group) and baseline (T0 for the intervention group and T4 for the control group)

**Figure SM1.** Individual differences in daily life gait performance during the home period (average of T2-T4) with respect to baseline (T0) (study phase 1) and during the home period (average of T7-T9) with respect to T4 (study phase 2). Dots (grey indicates conventional program and green Myosuit program) represent individual data points and black bars indicate the means with 95% confidence intervals.

**Supplementary material 2.** Secondary analysis primary outcome measure (regression analysis).

None of the explanatory variables were related to the change in walking time per day from baseline to the home period (*F*(8,19) = 1.49, *p* = 0.22, adjusted *R² =* 0.13) (see Table 1).

**Table SM2**. Regression coefficients from multiple linear regression model.^a^

| **Explanatory variable** | **Estimate** | **95% CI** | **p-value** |
| --- | --- | --- | --- |
| Age | 0.25 | [-0.37, 0.87] | 0.41 |
| Lesion level (Th) | 2.94 | [-22.39, 28.26] | 0.81 |
| Lesion level (C) | -0.80 | [-25.94, 24.34] | 0.95 |
| ASIA impairment scale | -12.00 | [-34.53, 10.54] | 0.28 |
| TSI | -0.08 | [-0.15, 0.00] | 0.05 |
| WISCI II | 2.20 | [-0.54, 4.94] | 0.11 |
| Somatosensation | -0.25 | [-0.70, 0.20] | 0.26 |
| Exosuit home use | 0.66 | [-0.12, 1.44] | 0.09 |

Abbreviations: Th, thoracal; C, cervical; ASIA, American Spinal Injury Association; TSI, time since injury; WISCI II, Walking Index for Spinal Cord Injury

^a^28 observations included (5 observations deleted because of missing data)

**Supplementary material 3.** Secondary analysis usability.

Figure SM2 showed the frequency and purpose of use of the control group following the Myosuit program (N=16). The average score on the SUS was 61 ± 15 at T6 and 49 ± 15 at T10, indicating marginal usability at T6 and not acceptable usability at T10. At T6, the average satisfaction was rated as 3.4 ± 0.5 (total D-QUEST score), with subscale scores assistive device 3.3 ± 0.7 and service 3.7 ± 0.4. The most frequently selected important items were: effectiveness (N=9 dissatisfied, N=7 satisfied), ease of use (N=11 dissatisfied, N=5 satisfied), and weight (N=8 dissatisfied, N=8 satisfied). After using the Myosuit at home (T10), Myosuit use was rated by 15 participants (one dropout during the home period). The average satisfaction with the Myosuit was rated as 3.3 ± 0.4 (total D-QUEST score), with subscale scores assistive device 3.0 ± 0.5 and service 3.8 ± 0.4. The most important items were: effectiveness (N=15 dissatisfied, N=0 satisfied), ease of use (N=12 dissatisfied, N=3 satisfied), and comfort (N=12 dissatisfied, N=3 satisfied).

**Figure SM2.** Myosuit use of the control group following the Myosuit program (second study phase). (A) Frequency of use per week. Grey lines represent individual use, and the dark green line indicates the mean use per week. (B) Activities during which the Myosuit was used.

**Supplementary material 4.** Individual differences in gait capacity outcomes between post-intervention and baseline.

**Figure SM3.** Individual differences in gait capacity outcomes between post-intervention (T5) with respect to baseline (T0). Minimal clinically important differences (MCIDs) are presented when available and are derived from the research of Forrest et al. (2014). (A) Preferred walking speed (MCID: 0.15 m/s), (B), maximum walking speed (MCID: 0.15 m/s), (C) walking distance (MCID: 36 m), and (D) SCI-FAP score. Dots (grey indicates conventional program and green Myosuit program) represent individual data points and black bars indicate the means with 95% confidence intervals.

**Supplementary material 5.** Secondary analyses gait capacity.

|  | Intervention group (Myosuit program)  n = 15 | | | Control group (Myosuit program)  n = 16 | | |
| --- | --- | --- | --- | --- | --- | --- |
|  | T0 | T5 | Difference^b^ | T5 | T10 | Difference^b^ |
| Preferred walking speed | 0.57 ± 0.27 | 0.66 ± 0.33 | 0.09 (0.01, 0.16) | 0.61 ± 0.28 | 0.58 ± 0.29 | -0.03 (-0.07, 0.02) |
| Maximum walking speed | 0.73 ± 0.36 | 0.80 ± 0.40 | 0.08 (-0.005, 0.16) | 0.74 ± 0.34 | 0.71 ± 0.36 | -0.03 (-0.08, 0.03) |
| Walking distance | 211 ± 132 | 240 ± 134 | 29 (6, 52) | 204 ± 108 | 209 ± 115 | 5 (-11, 21) |
| SCI-FAP | 287 ± 347 | 286 ± 349 | -1 (-9, 6) | 286 ± 503 | 284 ± 502 | -2 (-7, 4) |

**Table SM3.** Gait capacity measures.^a^

Abbreviations: SCI-FAP, Spinal cord injury functional ambulation profile

^a^ Presented as mean ± SD unless indicated otherwise

^b^ Presented mean (95% confidence interval): difference between post-intervention (T5 for the intervention group and T10 for the control group) and baseline (T0 for the intervention group and T5 for the control group)

**Supplementary material 6.** Secondary analyses quality of life.

The mean difference in gained QALYs between T10 and T5 was 0.06 ± 0.15 for the control group after following the Myosuit program. The cost per QALY was €207,980.
